# GH-secreting pituitary macroadenoma (acromegaly) associated with progressive dental malocclusion and refractory CPAP treatment

**DOI:** 10.1186/s13005-017-0140-6

**Published:** 2017-05-10

**Authors:** Jaume Miranda-Rius, Lluís Brunet-LLobet, Eduard Lahor-Soler, David de Dios-Miranda, Josep Anton Giménez-Rubio

**Affiliations:** 1 Department of Odontostomatology, Faculty of Medicine & Health Sciences, Universitat de Barcelona, Feixa Llarga, s/n, L’Hospitalet de Llobregat, 08907 Barcelona, Spain; 2 Division of Orthodontics and Paediatric Dentistry, Hospital Sant Joan de Déu, Universitat de Barcelona, Barcelona, Spain; 3 Bachelor of Science, Health Sciences Program, Universitat Autònoma de Barcelona, Barcelona, Spain; 4 Chief of Oral and Maxillofacial Department, Hospital Universitari Mútua de Terrassa, Universitat de Barcelona, Terrassa, Spain

**Keywords:** Dental malocclusion, CPAP mask user, Obstructive sleep apnea syndrome, Acromegaly, Pituitary adenoma

## Abstract

**Background:**

A link between progressive dental malocclusion, the use of a continuous positive airway pressure mask and GH-secreting pituitary macroadenoma (acromegaly) has not been previously reported. The present clinicopathological analysis stresses that tooth malposition should not be seen exclusively as a local process.

**Case presentation:**

A 62-year-old caucasian man with no relevant medical history reported difficulty chewing food and perceived voice alteration during his annual periodontal check-up. He also referred stiffness of the tongue, face, and submandibular area. The patient had been diagnosed with obstructive sleep apnea syndrome two years previously, since when he had worn a continuous positive airway pressure device during sleep. Exploration of the occlusion revealed significant changes: an atypical left lateral and anterior open bite with major buccoversion of teeth 33, 34, 35, 36. Inspection of the soft tissue revealed only macroglossia, although external palpation indicated a subcutaneous stiffness of the submandibular area. General analytical tests, including hormone profiles, and magnetic resonance imaging confirmed the diagnosis of acromegaly induced by a pituitary adenoma. Intrasellar tumor resection via transsphenoidal approach was performed. After surgery, the patient already noted a marked improvement of all symptoms associated with the acromegaly. Desaturation data also evolved favourably and the pulmonologist advised the patient to abandon the continuous positive airway pressure treatment.

**Conclusion:**

Progressive dental malocclusion may be associated with a systemic disease and the use of a nasal mask with premaxillary support may distort the diagnosis of acromegaly.

## Background

In the case described here, a dental malocclusion was the starting point for the identification of an intracranial neoplasia which was concealed by the presence of obstructive sleep apnea syndrome (OSAS) and by the use of a nasal mask for continuous positive airway pressure (CPAP) treatment. The clinicopathological analysis of this progressive dental malocclusion in a CPAP mask user shows that tooth malposition should not be seen exclusively as a local process.

## Case presentation

**Fig. 1:**
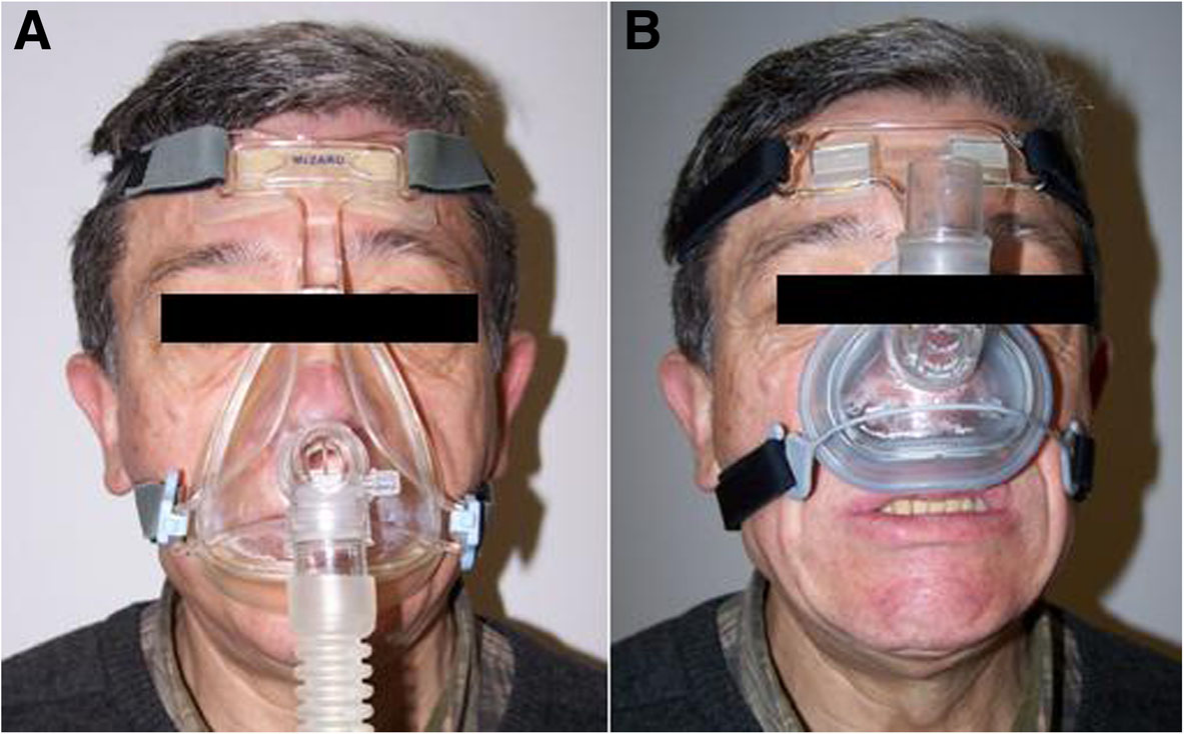
**a**) Oronasal (facial) mask. **b**) Nasal mask with support on the premaxillary area

A 62-year-old caucasian man with no relevant medical history reported difficulty chewing food and perceived voice alteration during his annual periodontal check-up. He also referred stiffness of the tongue, face, and submandibular area. The patient had been diagnosed with severe OSAS two years previously, since when he had worn a CPAP device during sleep. Polysomnography (PSG) showed an Apnea-Hypopnea Index (AHI) of 54.6. The lowest value of SaO_2_ (%) desaturation was 69, and it was below 90 for 43.5% of the total time asleep. At the first patient wore a mask that covered the mouth and nose with a flow of 9 cm H_2_O (Fig. 1a). However, after a control polysomnography at 14 months he was instructed to increase the flow pressure to 11 cm H_2_O, and for reasons of comfort he opted for an exclusively nasal mask. During the check-up with his periodontist, the mask was adjusted to assess the force exerted on the oral, dental and facial structures (Fig. 1b). The patient associated the changes in mastication with the pressure exerted by the last nasal mask on the area of the premaxilla and the upper incisors over recent months.

**Fig. 2:**
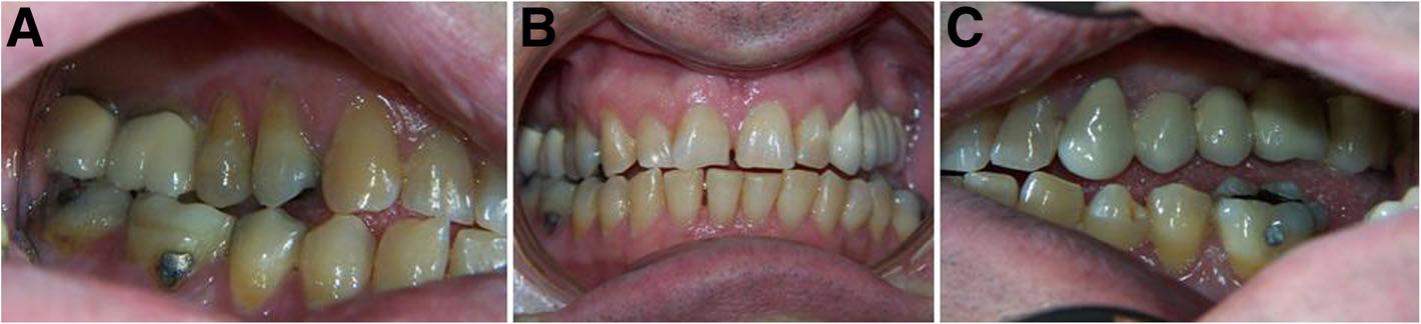
Clinical images. **a**) Right occlusion, showing a class III occlusion with loss of occlusal contacts due to the expansion caused by the macroglossia. **b**) Maximum intercuspidation (front view). Note the loss of contacts with a tendency towards open bite and the diastema in the anterior lower sector. **c**) Left occlusion, showing the posterior open bite associated with the growth and pressure of the tongue

Exploration of the occlusion with the aid of an orthodontist revealed significant changes. An atypical left lateral and anterior open bite was observed, with major buccoversion of teeth 33, 34, 35, 36, along with a slight diastema of the lower incisors, without tooth mobility (Fig. 2a,b,c).

**Fig. 3:**
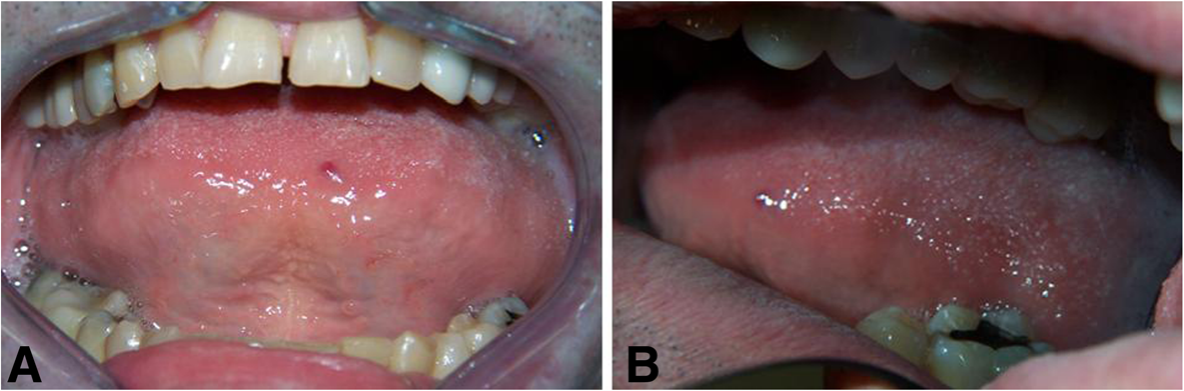
**a**) Clinical image of macroglossia. The tongue invades occlusal surfaces of the lower arch. **b**) Note the buccal version of teeth 35 and 36

**Fig. 4:**
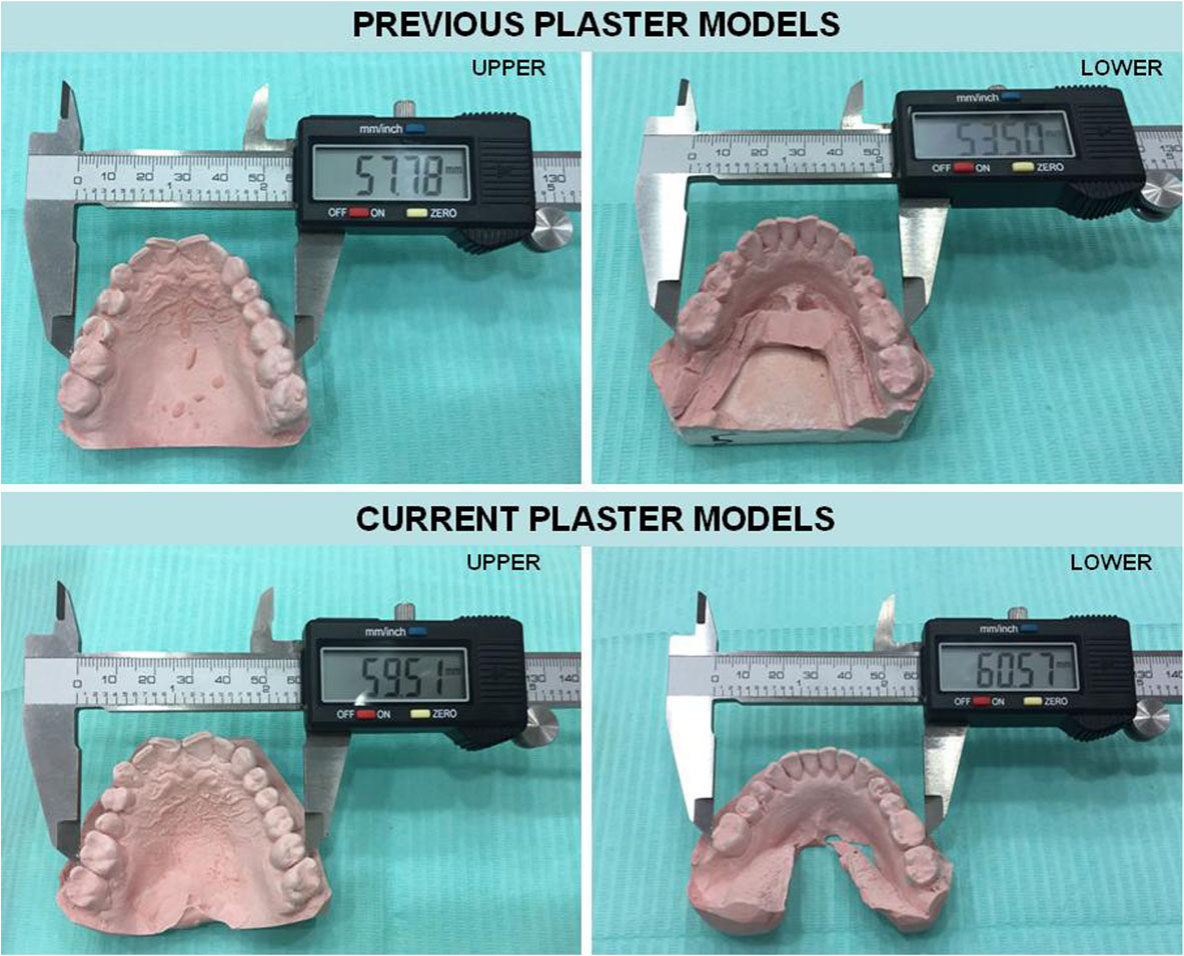
Measurement of the growth of the jaws in cross-sectional direction using a digital caliper in previous and current plaster models. Note the increases in the upper and lower distances between the teeth (2 mm and 7 mm respectively)

A soft tissue inspection revealed only macroglossia, with the tongue resting on the occlusal surfaces of the teeth of the lower arch (Fig. 3a,b). Fortunately, the availability of a set of study plaster models created five years previously allowed us to measure and accurately quantify the expansion of the jaws by using a digital caliper (Fig. 4).

**Fig. 5:**
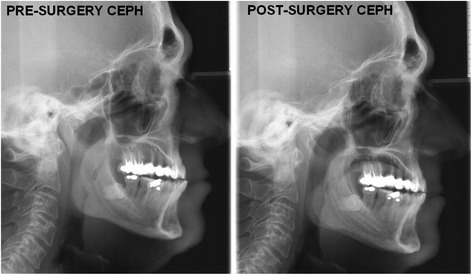
Comparison of lateral cephalometric x-ray previous to tumor resection and 12 months later. Note the normalization of the morphology of the sella turcica and the improvement in the inclination of the maxillary incisors

External palpation indicated a subcutaneous stiffness of the submandibular area. Orthopantomography was normal, although the lateral X-ray of the skull and cephalometric analysis showed abnormally high angles of inclination of the lower incisor with regard to the axis of the mandibular body and tooth plane (Fig. 5).

### Diagnosis and management

On the same day as the visit, orthopantomography and lateral cephalometry were performed, revealing a reduction of the upper airway with thickening of the soft tissue. General analytical tests were performed immediately, including hormone profiles. The extremely high values of growth hormone (11.8 ng/mL – normal range 0.02-1.23) and insulin-like growth factor 1 (780.2 ng/mL – normal range 15-246) confirmed the diagnosis of acromegaly and ruled out the rest of entities included in the differential diagnosis, such as pathological tooth migration, jaw expansive process, amyloidosis, hypothyroidism, rhinolalia and systemic voice disorders.

**Fig. 6:**
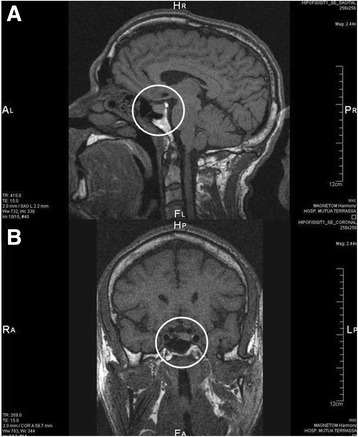
MRI study of the pituitary gland with IV contrast medium. Tomography in **a**) sagittal and **b**) coronal planes. Left intrasellar lesion measuring 11.5 × 9.5 mm touching the cavernous sinus wall. Note the normal glandular tissue on the right, the deformity of the floor of the sella, and the central position of the pituitary stalk

The patient was referred to an endocrinologist who requested a magnetic resonance imaging (MRI) of the pituitary gland with intravenous contrast. A significant growth of the left side of this gland (11.5x9.5 mm) was identified and the image was compatible with a pituitary macroadenoma (Fig. 6).

**Fig. 7:**
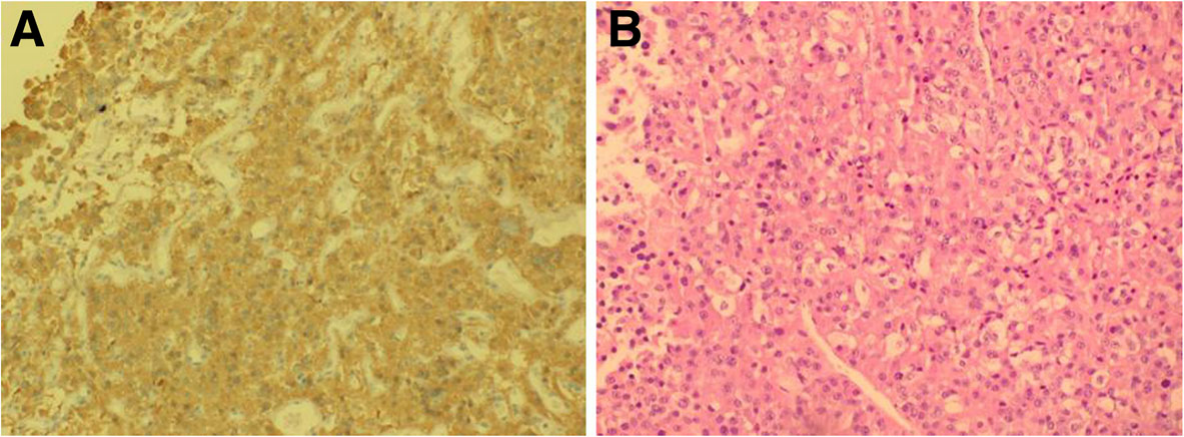
Microscopic appearance. **a**) Immunohistochemical study of GH indicating granular and diffuse staining patterns in the cytoplasm of tumor cells. **b**) Altered architecture with cells presenting moderate pleomorphism and abundant cytoplasm (H&E, original magnification 20×)

To complete the preoperative study, neuronavigation computed tomography with intravenous contrast was performed, which confirmed the left intrasellar tumor. Finally, the patient underwent tumor resection via a transsphenoidal approach, conducted by a neurosurgeon. Postoperative evolution was satisfactory and histopathological analysis confirmed the diagnosis of densely granulated somatotroph pituitary macroadenoma (Fig. 7).

A few days after surgery, the patient already noted a marked improvement of all symptoms associated with the acromegaly and decided not to resume CPAP treatment. In addition desaturation data also evolved favorably during follow-up: the lowest SaO_2_ (%) value was 87.60 and it was below 88% for only 0.02% of the total time asleep. Consequently, the pulmonologist advised the patient to abandon the CPAP. Within only two months, the analytical values showed a clear trend towards normalization: growth hormone (GH) 0.305 ng/mL and insulin-like growth factor 1 (IGF-1) 302.6 ng/mL.

## Discussion

Pituitary adenomas are benign neoplasms arising from cells of the anterior pituitary gland and constitute 10-15% of intracranial tumors. From the clinicopathological point of view, they are divided into as somatotroph adenomas, which produce growth hormone; lactotroph adenomas, which produce prolactin; thyrotroph adenomas, which produce thyroid-stimulating hormone (TSH); corticotroph adenomas, which produce adrenocorticotropic hormone ACTH); and gonadotroph adenomas, which produce gonadotropin [1]. It is extremely unusual for the dentist to suspect a somatotroph pituitary adenoma during a periodontal maintenance visit.

Patients with acromegaly are usually diagnosed late (>3 years) and at very advanced stages of the disease. In our case, the time to diagnosis was approximately two years; when OSAS was diagnosed, acromegaly should have been suspected. About 40% of cases of acromegaly are diagnosed by internists, and they may also be diagnosed by specialists in sleep obstructive disorders or ophthalmologists [2]. Acromegaly usually causes craniofacial abnormalities and indeed almost all cases present significant changes in oral and dental structures. The facial changes typically associated with acromegaly include an increasingly prominent forehead, growth of the nose and ears, thickening of the lips and the development of marked nasolabial folds. Patients also present mandibular prognathism, which may cause malocclusion and diastema [3, 4]. Macroglossia is common in these patients and contributes to the occurrence of OSAS. The voice is characteristically sonorous, associated with laryngeal hypertrophy and enlargement of the sinuses [5]. In view of the major impact of changes in the orofacial area and the dental occlusion, Herrmann et al concluded patients with acromegaly require good oral and maxillofacial examination in strict co-operation between endocrinologists, dentists and oral surgeons [6]. Kashyap et al. suggest that acromegaly should be considered in patients who develop malocclusion after adolescence [7].

The persistence of high levels of GH and IGF-1 in patients with acromegaly raises the mortality rate to 30% [2]. The factors that contribute to the increased mortality in these patients include cardiovascular disorders (60%), respiratory disorders with sleep apnea (25%), neoplasms and hyperglycemia or overt diabetes [8].

The treatment of GH-secreting pituitary adenoma aims to normalize the values of the altered biochemical markers, to eliminate or control the associated tumor without damaging the pituitary function and to eliminate the signs and symptoms [9]. Currently, treatment comprises a combination of medical and surgical therapy. Three types of drugs are available: somatostatin analogs (SSA) such as lanreotide, dopamine agonists such as cabergoline and, above all, GH receptor antagonists such as pegvisomant [10].

Although most clinical guidelines advocate surgery as first-line treatment [11], Fougner et al’s meta-analysis observed a trend towards the use of medical therapy prior to surgery [12]. One of the consensus groups on acromegaly recently recommended drug treatment combining a long-acting SSA and pegvisomant in patients in whom GH and IGF-1 levels did not decrease with somatostatin agonists alone (around 50% of cases) [13].

The cure rates achieved using transsphenoidal surgery differ significantly depending on the size of the tumor and the experience of the neurosurgeon. Success rates of around 78% in surgery for microadenomas (<1 cm) and around 50% in macroadenomas (> 1 cm) have been reported [13]. A recent study using data from the UK National Acromegaly Registry reported success rates between 20 to 40% with transsphenoidal surgery [14].

The association between acromegaly and OSAS has been reported previously [15, 16]. Seventy-three percent of patients with acromegaly present enlarged goiters and a high prevalence of type II diabetes [15, 17], but the size of the thyroid and the glucose metabolism are not sufficient to explain the association between acromegaly and OSAS. An excess of GH and IGF-1 lead to anatomical changes in craniofacial bones, soft tissues, and respiratory mucosa, so these patients often develop OSAS (as many as 80-90% in some series) day time narcolepsy, and cognitive impairment severe enough the quality of life. Acromegaly is also associated with “central apnea” which streams from alterations in the non-behavioral system controlling ventilation caused by the direct effects of GH/IGF-1 levels on the respiratory center [15]. Treatment with CPAP is effective in controlling sleep disorders in patients with acromegaly, although the results for the normalization of GH/IGF-1 and the improvement of OSAS are controversial [18–20]. In our case, the patient presented improvements in clinical symptoms and oxygen saturation levels after the intervention, coinciding with declining levels of GH/IGF-1.

Some authors have claimed that obesity is related to OSAS [21], but our patient had a body mass index (BMI) of 23. To rule out subclinical disease in patients suffering from sleep apnea, especially in those who are not overweight, an analytical hormonal profile is required.

Our study raises important questions about the application of different types of oronasal or nasal face masks for CPAP treatment and the presence of progressive dental malocclusion in these patients. To date, few studies have discussed the link between these two variables. The limited evidence available shows an association between prolonged use of the nasal mask and progressive occlusal and lateral variations in lateral and anterior sections, and suggests that the mask may also be the cause of open bite [22].

Pneumologists and sleep disorder specialists should be alert to the possibility of acromegaly in OSAS patients. Dental, oral and maxillofacial specialists should also be able to recognize specific local signs (not only tongue volume) that are suggestive of acromegaly. Patients who are candidates for treatment with CPAP should undergo a thorough anamnesis, especially if they have macroglossia associated with stiffness of facial tissues. It is important to ask patients about the growth of hands, feet, nose, and the sensation of distended and globular abdomen due to generalized visceromegaly. Patients with acromegaly may also experience growth in both prostate and colon leading to slow intestinal transit, constipation and difficulty urinating. Some studies show a higher prevalence of intestinal polyps and colon cancer [23].

In acromegaly, the trend towards mandibular growth involves protrusion and widening which, together with the macroglossia, causes the malocclusion and diastema observed. In addition, it is usually accompanied by a hypertrophy of the laryngeal tissues that may cause alterations in the voice [11]. All these changes in the mouth, teeth, face and voice were identified in our patient.

Although many patients with acromegaly are successfully operated on or treated and show improvement of their AHI, normalization is infrequent because many anatomical aspects are not reversed. However, the favorable postsurgical evolution of this patient suggests that the OSAS could be attributed to macroglossia, subcutaneous stiffness of the submandibular area, and hypertrophy of the laryngeal tissues secondary to acromegaly. Significantly, after the removal of the pituitary adenoma and the ensuing reduction in the volume of orofacial soft tissue next to the airway, even though a second polysomnography was not performed after surgery the OSAS improved markedly and the desaturation data evolved favorably, with the result that the patient was able to abandon CPAP treatment.

Twelve months after tumor resection, the lateral cephalometric x-ray revealed a normalization of the morphology of the sella turcica. It also showed an improvement in the inclination of the maxillary incisors (Fig. 5). These changes can probably be attributed to the reduction of the volume of the tongue and consequently to the fall in pressure exerted on the incisors. At present the patient reports far less difficulty speaking, chewing and swallowing.

## Conclusions

In summary, progressive dental malocclusion may be associated with a systemic disease, and the use of a nasal mask with premaxillary support may distort the diagnosis of acromegaly. Only by raising awareness of acromegaly among specialists in different areas can the currently unacceptable delay in its diagnosis be reduced. A protocol that includes an oral examination and a hormone study should be applied both in patients suffering from sleep apnea and in patients with atypical progressive malocclusion associated with macroglossia.

## Abbreviations

ACTH: Adrenocorticotropic hormone
AHI: Apnea-Hypopnea Index
BMI: Body mass index
CPAP: Continuous positive airway pressure
GH: Growth hormone
IGF-1: Insulin-like growth factor 1
MRI: Magnetic resonance imaging
OSAS: Obstructive sleep apnea syndrome
PSG: Polysomnography
SSA: Somatostatin analogs
TSH: Thyroid-stimulating hormone.
